# *miR-376c* promotes carcinogenesis and serves as a plasma marker for gastric carcinoma

**DOI:** 10.1371/journal.pone.0177346

**Published:** 2017-05-09

**Authors:** Pei-Shih Hung, Chin-Yau Chen, Wei-Ting Chen, Chen-Yu Kuo, Wen-Liang Fang, Kuo-Hung Huang, Peng-Chih Chiu, Su-Shun Lo

**Affiliations:** 1 Department of Education and Medical Research, National Yang-Ming University Hospital, Yilan, Taiwan; 2 Department of Surgery, National Yang-Ming University Hospital, Yilan, Taiwan; 3 Department of Medicine, National Yang-Ming University Hospital, Yilan, Taiwan; 4 Division of General Surgery, Veterans General Hospital–Taipei, Taipei, Taiwan; 5 Department of Dentistry, National Yang-Ming University Hospital, Yilan, Taiwan; 6 School of Medicine, National Yang-Ming University, Taipei, Taiwan; University of Navarra, SPAIN

## Abstract

Gastric carcinoma is highly prevalent throughout the world. Understanding the pathogenesis of this disease will benefit diagnosis and resolution. Studies show that miRNAs are involved in the tumorigenesis of gastric carcinoma. An initial screening followed by subsequent validation identified that *miR-376c* is up-regulated in gastric carcinoma tissue and the plasma of patients with the disease. In addition, the urinary level of *miR-376c* is also significantly increased in gastric carcinoma patients. The plasma *miR-376c* level was validated as a biomarker for gastric carcinoma, including early stage tumors. The induction of *miR-376c* was found to enrich the proliferation, migration and anchorage-independent growth of carcinoma cells and, furthermore, the repression of the expression of endogenous *miR-376c* was able to reduce such oncogenic phenotypes. *ARID4A* gene is a direct target of *miR-376c*. Knockdown of endogenous *ARID4A* increased the oncogenicity of carcinoma cells, while *ARID4A* was found to be drastically down-regulated in tumor tissue. Thus, expression levels of *miR-376c* and *ARID4A* mRNA tended to be opposing in tumor tissue. Our results demonstrate that *miR-376c* functions by suppressing *ARID4A* expression, which in turn enhances the oncogenicity of gastric carcinoma cells. It seems likely that the level of *miR-376c* in plasma and urine could act as invaluable markers for the detection of gastric carcinoma.

## Introduction

The International Agency for Research on Cancer (IARC) has reported that gastric carcinoma (GC) to be the fifth most common malignancy worldwide. More than 50% of GC cases occur in Eastern Asia, which includes China, Japan, Korea and Taiwan. In 2014, GC ranked as the seventh largest cause of cancer death among all malignancies and sixth highest cause of cancer death among the male population of Taiwan. Surgical resection is the principal treatment modality of GC [1–3]. In general, the 5-year survival rate of resectable GC, with three out of five cases being resectable, is about 45% after the curative surgical operation, which implies that about 50% of patients suffer from recurrence of the tumor at a later time. Although the 5-year survival rate of late-stage patients is much lower than that of early-stage patients, the 10-year survival rate of resectable early GC patients is higher than 90%. Therefore, an early diagnosis of the disease is critical for successful surgical interception of GC.

Gastric carcinogenesis is a multistep neoplastic process that involves many genetic and environmental factors [4–7]. Despite the fact that environmental factors play important roles in GC carcinogenesis, it is still important to identify both genetic susceptibility factors as well as the others factors that predispose a patient to early metastasis. It is also very important to validate the mechanisms that underlie the metastasis and recurrence of this malignancy. Unfortunately an early diagnosis of GC is not feasible for most GC patients due to a requirement for diagnostic confirmation by gastroscopy and the lack of convenient non-invasive biomarkers that can be used for routine population screening. It would be of great benefit to identify novel biomarkers that are both non-invasive and convenient that will allow the simple detection of GC; these would greatly aid early diagnosis.

MicroRNAs (miRNAs; miRs) are short, non-coding endogenous RNAs; they are involved in the regulation of gene expression, which they do by controlling mRNA translation. The function of miRNAs is to repress gene activity and they mainly do this by binding with imperfect complimentarity to conserved sites in the 3'-untranslated regions (3'-UTRs) of a given gene, which blocks the translation of the mRNA transcript into protein [8]. Previous studies have reported that aberrant miRNA expression contributes to the neoplastic process in many types of cancers including GC, either via the control of oncogenes or via the control of tumor suppressors [9–12]. Research, including ours, has shown that *miR-21*, the *miR-17-92* cluster, *miR-146a*, *miR-200*, *miR-370* and a number of other miRNAs are up-regulated in GC, which means that they may act as oncogenic miRNAs [9, 13–17]. Circulating miRNAs have been found to be stable in the bio-fluids of cancer patients and many reports have demonstrated that these circulating miRNAs can indeed be used as biomarkers to identify and monitor a variety of cancers [18–21]. In addition, urine samples, which contain fewer proteins, seem to be better at preserving RNA than blood and this is becoming an important body fluid for the development of RNA biomarkers [22].

In our previous miRNA microarray screening [9], *miR-376a* and *miR-376c* were found to be up-regulated in GC tissue samples. Studies have shown that the *miR-376* family is likely to play crucial roles in tumorigenesis. In human cervical and liver cancer cell lines, *miR-376a* has been shown to directly target the matrix extracellular phosphoglycoprotein gene that reduces G2 arrest and sensitizes cells to DNA damage-induced killing [23]. Furthermore, *miR-376a* overexpression has been found to significantly inhibit the arsenic trioxide-induced apoptosis of retinoblastoma cells [24]. It has also been reported that *miR-376c* targets activin receptor-like kinase 7 and that this enhances the survival of ovarian cancer cells [2]. Using a K-ras transgenic rats model, the serum level of *miR-376a* was suggested as a potential novel pre-clinical biomarker for pancreatic ductal carcinoma [25]. In addition, serum *miR-376c* level has been strongly associated with the poor differentiation of GC [26]. Circulating *miR-376c* has also been shown to be conspicuously up-regulated in the plasma of breast cancer patients and this miRNA seems to have the potential for being developed as part of multi-marker blood-based tests for the early detection of breast cancer [27]. These reports when taken together imply that *miR-376a* and *miR-376c* are likely to be important tumor markers.

The human *ARID4A* gene belongs to the AT-rich interactive domain (ARID) gene family, which consists of 15 genes. All ARID proteins bind to DNA either at AT-rich sites or in a non-specific manner. The *ARID* gene family can be further divided into seven distinct subfamilies, namely the *ARID1*, *ARID2*, *ARID3*, *ARID4*, *ARID5*, *JARID1* and *JARID2* subfamilies [28]. Aberrant expression of ARID proteins is a fairly common feature of tumor cells [29]. Cancer genome sequencing has identified the presence of recurrent somatic mutations that inactivate *ARID1A* in a various neoplasms including GC [28, 30–32]. *ARID4A* is known to be a tumor suppressor in a variety of malignancies [33–35]. However, there was no report up to the present has ever explored *ARID4A* and its relationship with GC.

In this study, we demonstrated that *miR-376c* is up-regulated in GC tissue as well as the plasma and urine from GC patients. The present of *miR-376c* in plasma could be a potential novel clinical diagnostic biomarker for early stage GC. In addition, *miR-376c* was found to able to enhance the oncogenicity of GC cells via targeting of ARID4A.

## Materials and methods

### GC tissue, plasma and urine samples

Resected GC tumor tissue samples together with paired non-cancerous mucosa (NCM) samples were harvested from 35 patients (group 1 in Table 1) who underwent curative resection of a previously untreated GC in National Yang-Ming University Hospital, between 2013 and 2015. About 0.5cm^3^ of the resected tissue was ground up and total RNA isolated from the lysate. Pre-surgical plasma samples of GC subgroup were obtained from 65 patients who were diagnosed GC (group 2 in Table 1) between 2013 and 2016. The control plasma samples were obtained from outpatients (group 3 in Table 1) who underwent gastroscopic examination between 2015 and 2016 to exclude the presence of neoplastic lesion in the upper gastrointestinal (GI) tract. Each sample from the above subjects consisted of 5 ml of whole blood that was collected in a heparin-coated tube [9]. Furthermore, both pre-surgical plasma and post-surgical plasma, sampled later than 1 month after surgery, were obtained from eight patients who were part of group 2. Urine samples were also collected from the patients who formed groups 2 and 3. The age of subjects ranges from 20 to 94 years old. Patients having previous history of GC or the presence of GI neoplasms other than GC were excluded for analysis. This study was approved by a hospital institute review board (Approval No.: 2013-B003 and 2014-A019) and all subjects provided written informed consent before the sampling took place.

**Table 1:** Clinical parameters of the subjects.

	Group 1 (*n* = 35)	Group 2 (*n* = 65)	Group 3 (*n* = 108)
	GC tissue pair individuals	GC plasma sample individuals	Control plasma individuals
**Sex (Male/Female)**	23/12	47/18	67/41
**Age (Years; Mean ±SE)**	69.3 ± 2.2	67.7 ± 1.5	56.1 ± 1.5
	(33–87)	(33–94)	(20–89)
**TNM staging**			
**T1**	6	24	
**T2-T4**	29	41	
**N0**	15	33	
**N1**	6	9	
**N2**	14	23	
**Stage I**	8	30	
**Stage II**	9	14	
**Stage III**	14	16	
**Stage IV**	4	5	

No gender difference was noted between GC patients and control individuals (Fisher’s exact test; detailed analysis not shown), but control individuals were younger than GC patients (*p*<0.0001, un-paired *t*-test).

### Reagents

Chemically modified *miR-376* mimics (Cat. No.: MC11031 and MC12260) and an appropriate scrambled control (Cat. No.: 4464058), as well as *miR-376* locked nucleic acid (LNA) blockers (Cat. No.: MH11031 and MH12260) and an appropriate scrambled control (Cat. No.: 4464076) were purchased from Applied Biosystems (Thermo Fisher Scientific, Waltham, MA, USA). A small interference oligonucleotide, si*ARID4A* (Cat. No.: sc-6290), which was used to knock down *ARID4A* gene expression, as well as a control oligonucleotide (siControl; Cat. No.: sc-37007), were both purchased from Santa Cruz Biotech (Santa Cruz, CA, USA). TransFectin^TM^ (Cat. No.:170–3351, Bio-Rad, Hercules, CA, USA) lipid reagent was used for transfection. Unless specified otherwise, all other reagents were purchased from Sigma-Aldrich (St Louise, MO, USA).

### Quantitative RT-PCR (qRT-PCR)

Total RNA was purified using TRI reagent^®^. Applied Biosystems^®^ TaqMan MicroRNA Reverse Transcription Kits (Thermo Fisher Scientific) and TaqMan^®^ MicroRNA Assay Kits (Thermo Fisher Scientific) were used to detect the expression of *miR-376a* (Cat. No.: 000565) and *miR-376c* (Cat. No.: 002122). These reactions were carried out using iQ5 real-time PCR detection systems (Bio-Rad). *U6B* (Cat. No.: 001093), *miR-16* (Cat. No.: 000391) or *miR-1228* (Cat. No.: 002919) were used as controls for the analysis of cell, tissue and plasma samples [36, 37]. The mRNA expression levels of *ARID4A* (Cat. No.: Hs00243140_m1) and glyceraldehyde 3-phosphate dehydrogenase (*GAPDH*, internal control, Cat. No.: Hs02758991_g1) were analyzed using the Applied Biosystems^®^ TaqManq PCR System (Thermo Fisher Scientific). The mean threshold cycle (ΔCt) was achieved by normalization of the tested miRNA or mRNA signal against an internal control. The ΔΔCt was used to measure the relative changes in expression between tumor and NCM tissue samples and between the various experimental groups [9]. A negative control without a template was run in parallel to assess the overall specificity of the reaction. The 2^ΔΔCt^ values were used to represent the fold changes in miRNA or mRNA expression.

### Cell culture and proliferation assays

SCM-1 cell line originally established from a Taiwanese GC patient was grown in PRMI-1640 medium supplemented with 10% heat-inactivated fetal bovine serum (FBS, Biological Industries, Kibbutz Beit Haemek, Israel) at 37°C in a humidified atmosphere of 5% CO_2_ [9, 38]. The cell line was passaged at a density ratio of 1:5 when 80% confluence was reached [9]. Cell proliferation was analyzed by the MTT assay or by counting of cell numbers directly. For the MTT assay, the cells were seeded at 1000 cells/well using 96-well plates in triplicate. Growth curves were drawn using the values for OD_570nm_. For the cell number counts, the cells were seeded at 5000 cells/well using 48-well plates in triplicate. Cell growth curves were drawn based on the total number of cells present when counted at different time points.

### Transwell migration assay

Transwells (Corning, Acton, MA, USA) were pre-coated with fibronectin as a chemotactic attractor to induce cell migration. A total of 1x10^5^ cells were seeded into each Transwell containing serum-free medium in the upper chamber. Then 1 μM hydroxyurea was added to arrest cellular proliferation. After 24 hours, the migrated cells on the lower surface of membrane were stained with Hoechst 33258 (Thermo Fisher Scientific) and counted under fluorescence microscopy.

### Anchorage-independent colony forming assay

Using 6-well plates, lower agar layer was created using a 1:1 mixture of 1.8% agarose and 30% FBS. Next, an upper agar layer was formed using a 1:1 mixture of methylcellulose and 30% FBS such that it covered the lower agar layer. Then, 1x10^5^ cells were seeded into upper layer and the plates cultivated for 7 days. Finally, the colonies present on the plates were stained with 0.05% crystal violet and those colonies with a diameter larger than 50 μm in 15 randomly selected fields were counted using an inverted microscope.

### Reporter constructs and activity assay

The 3′-UTR sequence of the *ARID4A* gene which contains predicted *miR-376c* binding site was cloned into the pMIR-REPORTER vector (Applied Biosystems) to generate the reporter construct designated pMIR-ARID4A. The plasmid pRL-TL, which expresses the renilla luciferase gene, was used as a transfection control. After co-transfecting of *miR-376c* mimic with pMIR-REPORTER (vector alone; VA) or pMIR-ARID4A, as well as pRL-TL for 24 hours, the lysate were collected and assayed in accordance with the protocol of Dual-Luciferase^®^ reporter assay system (Promega, WI, USA). Firefly luciferase activity was normalized against renilla luciferase activity to indicate the reporter activity.

### Western blot analysis

A total of 30 μg of protein from the various whole cell lysates were resolved by electrophoresis using a 10% denaturing polyacrylamide gel; the approach followed previously described protocols [9]. Primary antibodies against ARID4A (Cat. No.: sc-81640), GAPDH (Cat. No.: sc-47724) and α-Tubulin (Cat. No.: sc-5286) as well as appropriate secondary antibodies were purchased from Santa Cruz Biotech. The protein signals were detected using a Western Lightning Chemiluminescence Reagent Plus Kit (Perkin-Elmer, Wellesley, MA, USA) and a Chemi-Smart 3000 image acquisition system (Viber Lourmat, Collégien, France).

### Statistics

The Fisher’s exact test, Mann–Whitney test, *t*-test, two-way ANOVA, linear regression analysis, receiver operating characteristic (ROC) analysis, and various bioinformatic modules were used for the statistical analysis. The results were considered to be statistically different when *p*< 0.05.

## Results

### *miR-376a* and *miR-376c* are up-regulated in GC

**Fig 1:**
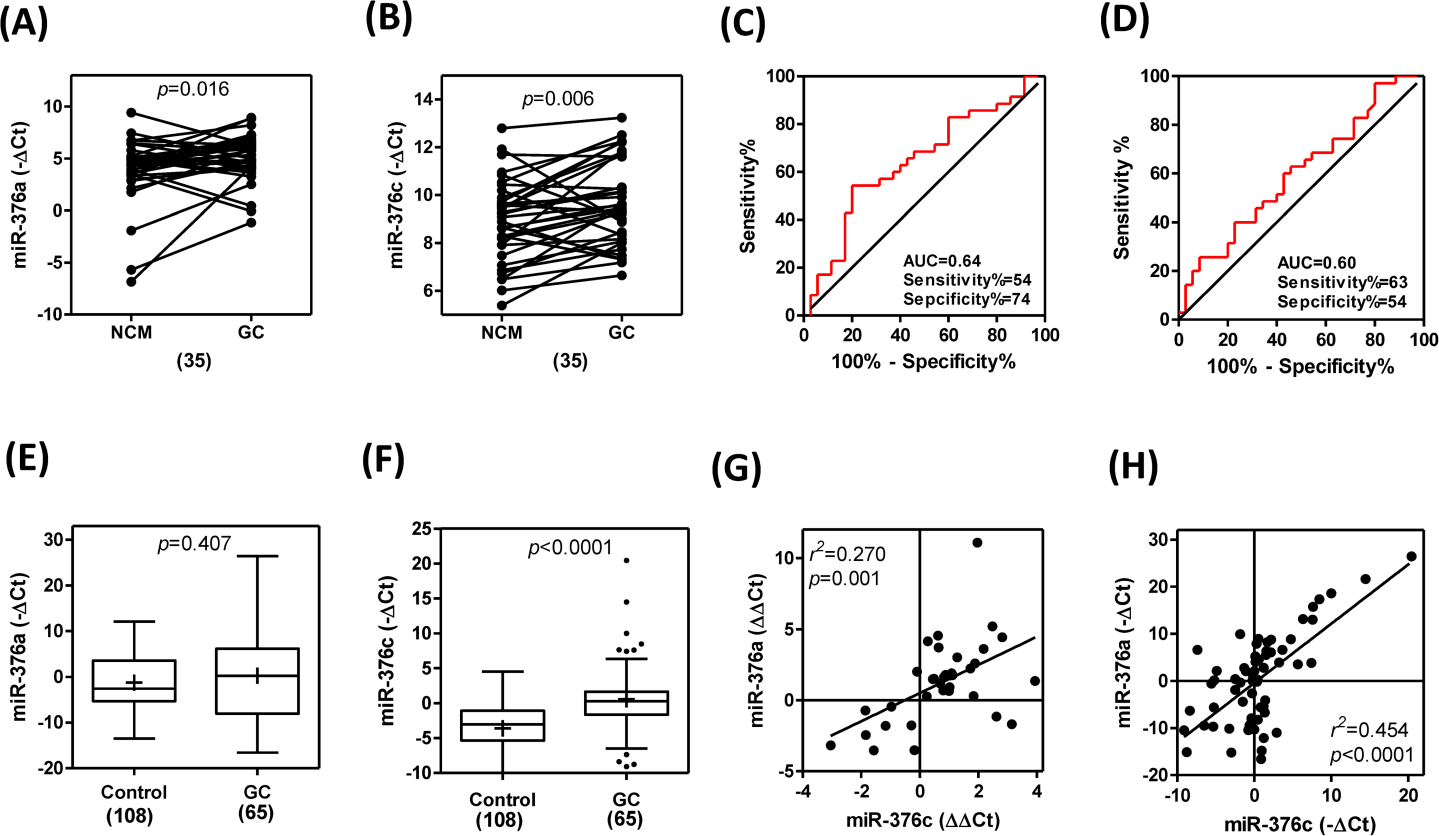
*miR-376a* and *miR-376c* in GC tissue and plasma. (A, B) Before-and-after plot. The–ΔCt of *miR-376a* (A) and *miR-376c* (B) in 35 pairs of GC and NCM. Paired *t*-test. (C, D) ROC analysis of *miR-376a* (C) and *miR-376c* (D).AUC, area under curve. (E, F) Whisker plot. The–ΔCt of *miR-376a* (E) and *miR-376c* (F) in plasma samples from 108 healthy control donors and 65 GC patients. +, mean value. Numbers within parenthesis, numbers of tissue pairs or cases. Un-paired *t*-test. (G, H) Linear regression analysis between the expression of *miR-376a* and *miR-376c* in GC tissues (G) and the expression of *miR-376a* and *miR-376c* in the patients’ plasma (H).

The expression of *miR-376a* and *miR-376c* in 35 paired GC and NCM tissue samples were analyzed. Both *miR-376a* and *miR-376c* were significantly up-regulated in the GC tissue compared to the NCM tissue (Fig 1A and 1B). ROC analyses showed that the expression levels of *miR-376a* and *miR-376c* in the GC tissue had predictive powers of 0.64 and 0.60, respectively, when distinguishing GC tissue from NCM tissue (Fig 1C and 1D) using area under curve (AUC) analysis. Although no statistically significant difference was found between the GC patients and control individuals for plasma *miR-376a* levels (Fig 1E), the amount of *miR-376c* in plasma was significantly higher in GC patients compared to control healthy individuals (Fig 1F). A positive correlation between the expression levels of *miR-376a* and *miR-376c* in the tissue (Fig 1G) and plasma (Fig 1H) of GC patients was found.

**Fig 2:**
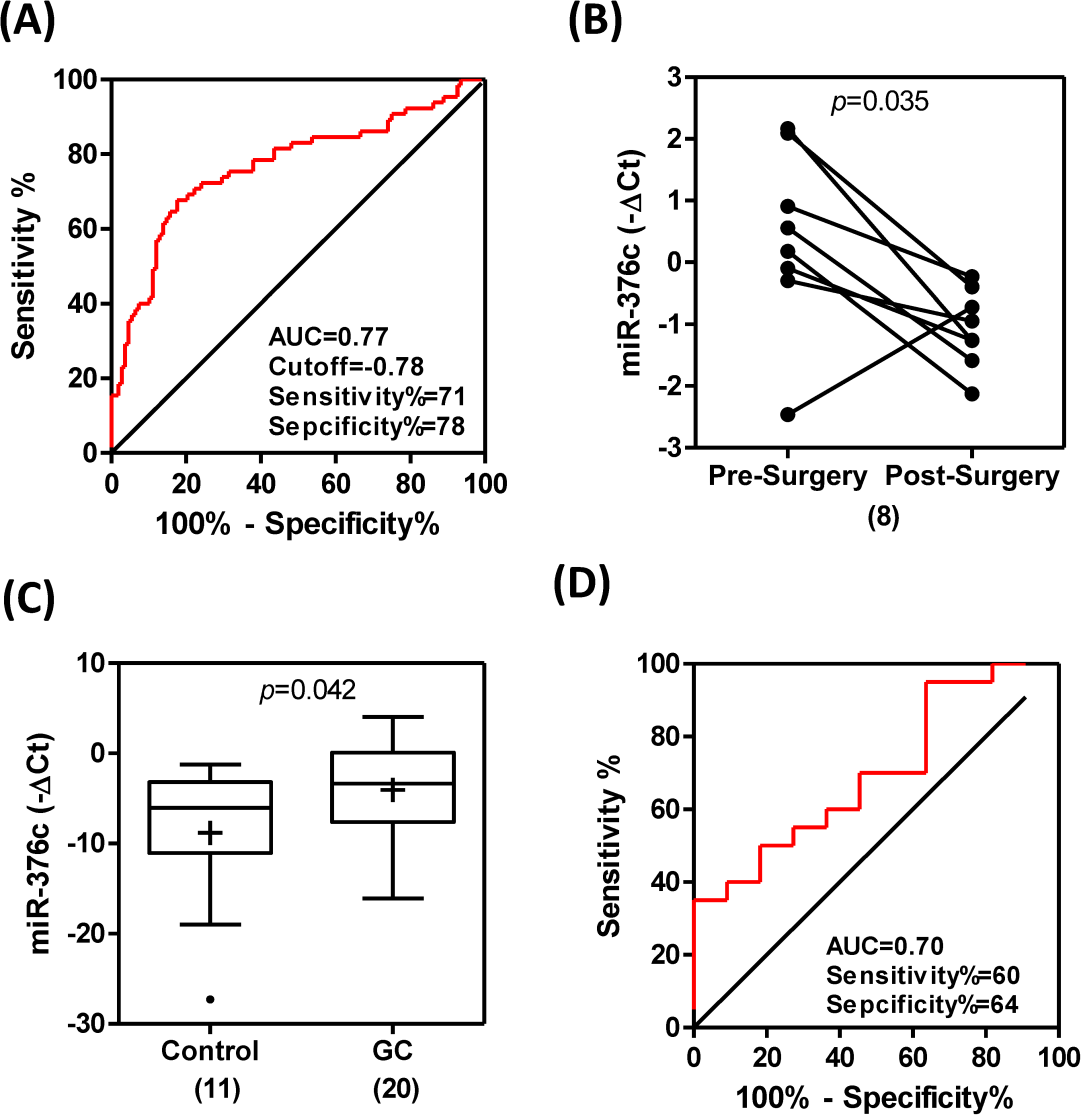
Plasma and urinary *miR-376c* in GC. (A) ROC analysis of plasma *miR-376c*. (B) Before-and-after plot. Changes of *miR-376c* in eight paired pre-surgical and post-surgical samples. Paired *t*-test. (C) Whiskers plot. Urinary *miR-376c* in 11 healthy controls and 20 GC patients. +, mean value. Numbers within parenthesis, numbers of cases. Un-paired *t*-test. (D) ROC analysis of urinary *miR-376c*. AUC, area under curve.

The ROC analyses indicated that plasma level of *miR-376c* in GC patients had a predictive power of 0.77 when separating GC patients from the control population (Fig 2A). Among the eight GC patients who had both pre-surgical and post-surgical plasma samples available, it was noted that there was a reduction in the plasma levels of *miR-376c* after tumor resection (Fig 2B). In addition, it was also found that the urinary levels of *miR-376c* were also significantly higher in GC patients compared to the control population (Fig 2C). The prediction power of urinary *miR-376c* with respect to GC was found to be about 0.70 (Fig 2D).

### Plasma *miR-376c* is up-regulated in early stage GC

**Fig 3:**
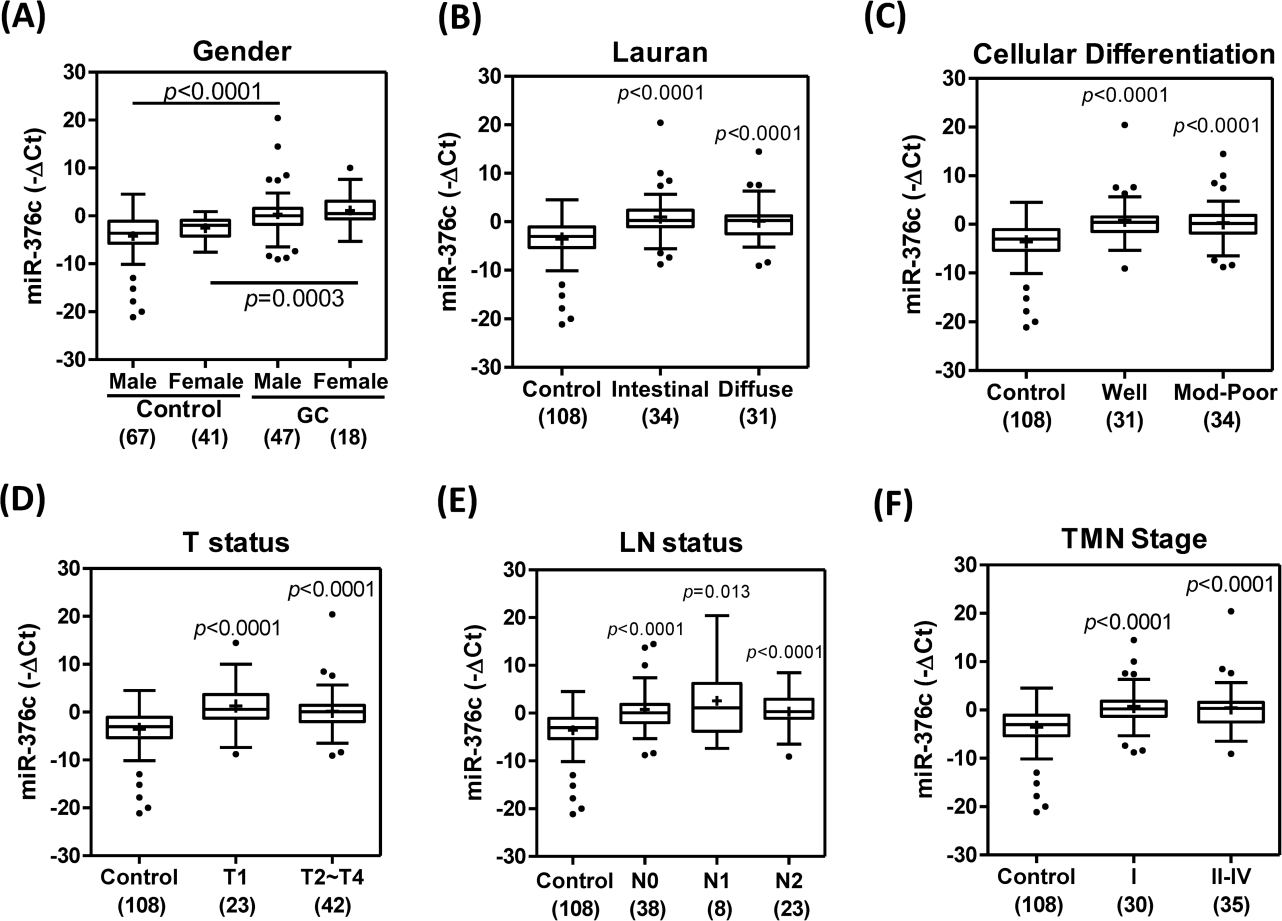
Plasma level of *miR-376c* and clinicopathological parameters. Whisker plots. (A) Gender. (B) Lauren classification. (C) Cellular differentiation. (D) Tumor size. (E) Nodal status. (F) Clinical stage. +, mean value. Numbers within parenthesis, numbers of tissue pairs or cases. Un-paired *t*-test.

We investigated the plasma levels of *miR-376c* in GC patients relative to the control individuals according to the clinicopathological features of the disease. The plasma levels of *miR-376c* were up-regulated in both male patients and female patients relative to controls of the same gender (Fig 3A). In addition, patients with intestinal type and well differentiated GC tumors had increased levels of *miR-376c* compared to controls (Fig 3B and 3C). It should be noted that plasma levels of *miR-376c* were also up-regulated in T1, N0 and Stage I patients (Fig 3D–3F). When tumors became more poorly differentiated, became larger, underwent nodal metastasis or underwent progression to a more advanced clinical state, the plasma level of *miR-376c* was found not to increase further (Fig 3B–3F). Thus, our findings imply that the up-regulation of *miR-376c* in plasma occurs during the early establishment stage of human GC tumorigenesis and that the plasma of *miR-376c* level does not seem to reflect either tumor aggressiveness or tumor burden.

As the average age of control individuals was younger than that of GC patients for more than ten years (Table 1), concerns were raised to wonder that the age difference might underlie the discrepancies of *miR-376c* in plasma. We thus divided group 2 and group 3 subjects into younger and older subgroups according to the median ages. The analysis showed that the plasma *miR-376c* in younger patients was higher than that in controls (S1A Fig). In addition, the plasma *miR-376c* in patients was also higher than that in older controls (S1B Fig). It is unlikely that age may confound the plasma *miR-376c* across sample groups.

### *miR-376c* promotes the oncogenicicity of the SCM-1 cell line

**Fig 4:**
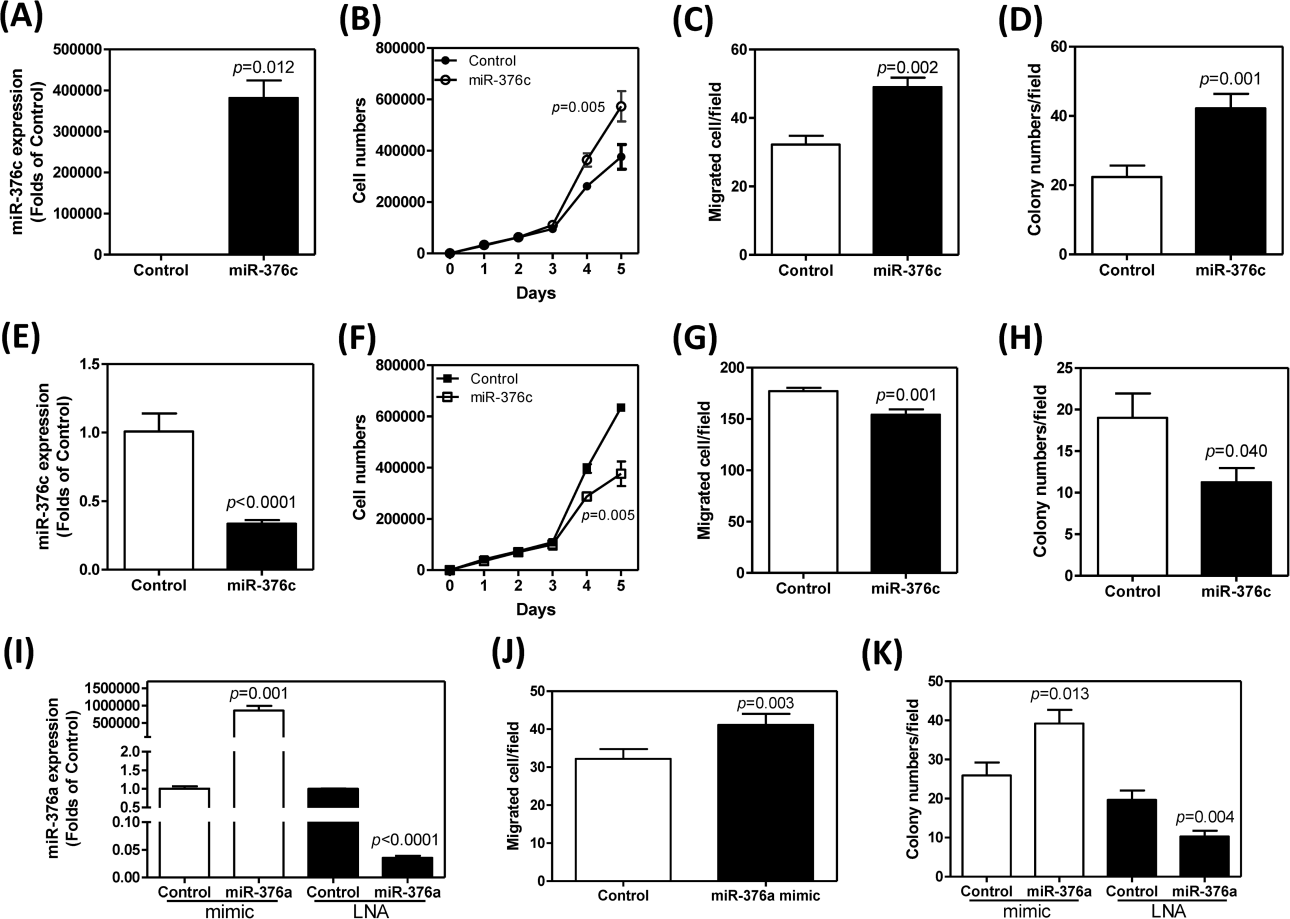
*miR-376*expression and the oncogenic phenotypes of SCM-1 cells. (A-H) *miR-376c*. (I-K) *miR-376a*. (A-D) Cells transfected with *miR-376c* mimic. (E-H) Cells transfected with *miR-376c* LNA. (I-K) Cells transfected with *miR-376a* mimic or *miR-376a* LNA. (A, E, I) qRT-PCR analysis to detect the expression of *miR-376*; Mann-Whitney test. (B, F). Proliferation assay; Two-way ANOVA test. (C, G, J) Migration assay; Mann-Whitney test. (D, H, K). Anchorage-independent growth; Mann-Whitney test. Each assay is independently carried out for three times. Data shown are mean ± SE in one representative assay.

The molecular functions of *miR-376c* were analyzed by carrying out transient transfections with*miR-376c* mimic and *miR-376c* blocker. At 24 hours after transfecting with mimic, *miR-376c* was found to be up-regulated in SCM-1 cells (Fig 4A). Exogenous *miR-376c* expression seemed to have significantly increased the proliferation (Fig 4B), migration (Fig 4C) and anchorage-independent colony formation (Fig 4D) of SCM-1 cells. By way of contrast, at 24 hours after transfecting with blocker, there was a decreased in the endogenous expression of *miR-376c* in SCM-1 cells (Fig 4E). Furthermore, in the presence of blocker, there were also a decrease in proliferation (Fig 4F), migration (Fig 4G) and anchorage-independent colony formation (Fig 4H), all relative to the control.

The molecular functions of *miR-376a* were also addressed by transfecting *miR-376a* mimic and *miR-376a* LNA blocker. Treatment with *miR-376a* mimic significantly increased exogenous *miR-376a* expression level of the SCM-1 cell line (Fig 4I, Lt) as well as increasing migration (Fig 4J) and anchorage-independent colony formation by this cell line (Fig 4K, Lt). However, treatment with *miR-376a* LNA blocker decreased the level of endogenous expression *of miR-376a* (Fig 4I, Rt) together with bringing about a reduction in the anchorage-independent colony formation of SCM-1 cells (Fig 4K, Rt).

### *miR-376c* negatively regulates *ARID4A* expression in SCM-1 cells

**Fig 5:**
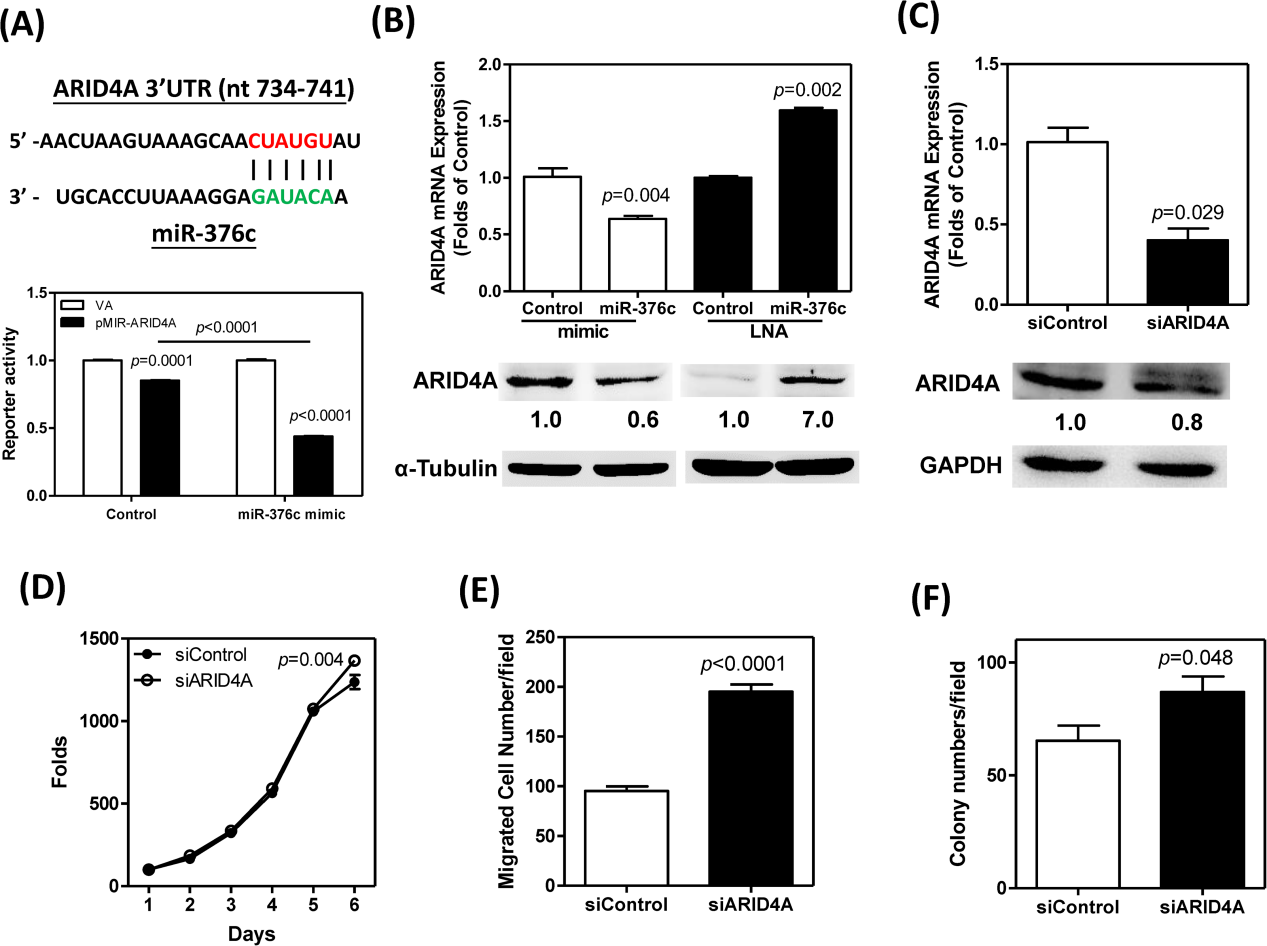
*miR-376c* decreases the expression of *ARID4A* and *ARID4A* acts as a suppressor in SCM-1 cells. (A) Upper, the predicted target sequence present in the *ARID4A* 3’UTR (red) and the seed sequence (green) of *miR-376c*. Lower, reporter activity. VA, control reporter. Decreased activities are noted in pMIR-ARID4A reporter in relation to VA reporter. The decrease is particularly drastic with the treatment of *miR-376c* mimic. (B) Cells transfected with *miR-376c* mimic or *miR-376c* LNA. (C) Cells transfected with si*ARID4A*. (B, C) *ARID4A* expression. Upper, qRT-PCR analysis to detect mRNA expression; Lower, Western blot analysis to detect protein expression. The numbers are normalized values. The pictures are from one representative analysis. (D-F) Phenotypic changes following the knockdown of *ARID4A*. (D) Proliferation. (E) Migration. (F) Anchorage-independent colony formation. Mann-Whitney test or two-way ANOVA. Each analysis is independently repeated twice. Data shown are mean ± SE in one representative analysis.

The TargetScan (v7.1) *in silico* module predicted that *ARID4A* is a putative target of *miR-376c*. The alignment of *miR-376c* and the 3’-UTR of the *ARID4A* gene, which contains the target sequences of *miR-376c*, is illustrated in the Upper panel of Fig 5A. The activity of pMIR-ARID4A reporter was slightly lower than control reporter in cells transfected with control oligonucleotide. After transfection with the *miR-376c* mimic for 24 hours, the presence of exogenous *miR-376c* was able to further suppress the activity of pMIR-ARID4A reporter (Fig 5A, Lower). In addition, the expression of *ARID4A* mRNA and protein in SCM-1 cells were also suppressed (Fig 5B, Lt). However, the reduction in *miR-376c* expression brought about by treatment with *miR-376c* LNA blocker increased the expression of *ARID4A* at both the mRNA and protein level (Fig 5B, Rt). To elucidate the functions of ARID4A, si*ARID4A* oligonucleotide and its equivalent scrambled control (siControl) were transiently transfected into SCM-1 cells. At 24 to 48 hours after transfecting with the active siRNA, the levels of *ARID4A* mRNA and protein expression were found to have decreased (Fig 5C). This decrease in endogenous ARID4A expression seemed to be associated with a slight up-regulation in cell proliferation (Fig 5D), and this in turn brought about a resultant up-regulation of migration (Fig 5E) and anchorage-independent colony formation (Fig 5F) by SCM-1 cells.

### Down-regulation of *ARID4A* expression in GC tumors

**Fig 6:**
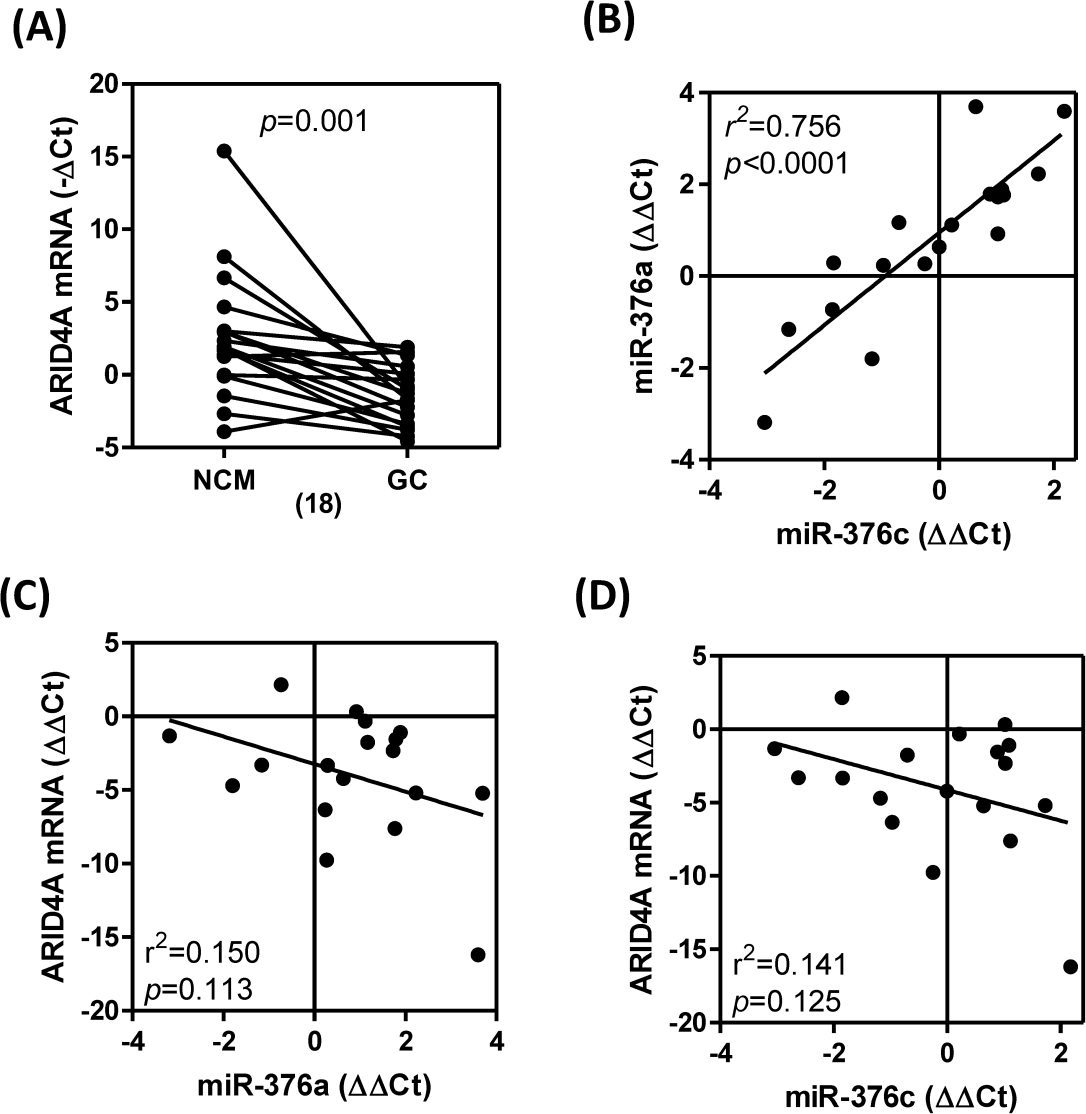
Down-regulation of *ARID4A* expression in GC. Before-and-after blot. *ARID4A* mRNA expression in 18 pairs of GC and NCM tissues. Numbers within parenthesis, numbers of tissue pairs. Paired *t*-test. (B-D) Linear regression analysis in 18 pairs of GC and NCM tissues. (B) The analysis between the expression of *miR-376a* and *miR-376c*. (C) The analysis between the expression of *miR-376a* and the expression of *ARID4A* mRNA. (D) The analysis between the expression of *miR-376c* expression and the expression of *ARID4A* mRNA.

*ARID4A* mRNA expression was analyzed in randomly selected group 1 sample pairs to try to unravel the reasons behind the drastic down-regulation of *ARID4A* expression in the GC tumors (16/18, 88.9%; Fig 6A). The increased expression of *miR-376a* and *miR-376c* in this subset of tumors was highly correlated with a decrease in *ARID4A* expression (Fig 6B) and this was consistent with the findings outlined in Fig 1G. Although the correlation between expression of *miR-376a* and expression of *ARID4A* (Fig 6C) or between expression of *miR-376c* and expression of *ARID4A* (Fig 6D) was not significant, the profiling was able to demonstrate the presence of a trend towards a reverse correlation between *miR-376* expression and *ARID4A* expression.

The loss of *ARID1A* is one of the key factors that confounds GC survival [32]. According to a large scale bioinformatics study encompassing 4,623 tumors from multiple anatomical sites [39], *ARID4A* is one of the genes found within the chromatin regulatory element group that exhibits potential driver alterations in one or more cancer types (detailed analysis not shown). Although the average mutation frequency of *ARID4A* in all tumors is only 1.1%, the mutation frequency of *ARID4A* in GC is 13.6% (3/22); this is despite the fact that the mutation frequency of *ARID1A* compared to *ARID4A* is much higher (27.3%; 6/22) in tumors. Furthermore, in a TCGA-based cBioPortal database (http://www.cbioportal.org/), it has also been revealed that there is much more frequent *ARID1A* mutation (28%; 111/393) than *ARID4A* (6%; 28/393). However, an analysis of the 478 GC patients in the cBioPortal showed an immensely lower abundance of the *ARID4A* transcript (expression average value~800) relative to the *ARID1A* transcript (expression average value ~3700) (S2A Fig). Finally, the RNA-sequencing results from EMBL-EBI (http://www.ebi.ac.uk/) also show that there is a lower amount of the *ARID4A* transcript than *ARID1A* transcript present in eleven GC cell lines (S2B Fig). Taking all this informed together, it seems likely that the expression of *ARID4A* is low in GC.

The searching of cBioPortal database revealed the absence of *miR-376c* mutation in GC and the rare occurrence of *miR-376c* up-regulation in less than 3% GC tumors (S1 Table).

## Discussion

In our previous study, we found that *miR-370* was up-regulated and that it contributed to the progression of GC [9]. As an extension of our previous work, this study has confirmed that *miR-376a* and *miR-376c* are up-regulated in GC tissue. The *miR-376* family is located on 14q32 and consists of several related miRNAs, including *miR-376a1-a2*, *miR-376b*, *miR-376c* (previously named as *miR-368*), *miR-654*, *miR-B1*, *miR-B2* and so on. These miRNAs have highly similar sequences [40]. Studies have revealed that the *miR-376* family plays important roles in cancer progression. It had been reported that *miR-376b* and *miR-654* are down-regulated in human lung cancer [41, 42]. By way of contrast, *miR-376a* and *miR-376c* are up-regulated in breast cancer and *miR-376a* may be useful as a breast cancer detection marker [27, 43]. This study identifies for the first time that *miR-376c* is up-regulated in both primary GC and in the plasma of GC patients. The findings of increased *miR-376c* in the plasma of GC patients are in agreement with a previous study [26]. Furthermore, since the plasma level of *miR-376c* decreases after tumor resection, it is likely that the *miR-376c* found in the plasma originates from the tumor. However, the discrepancy between our cohort and cBioPortal database in the frequency of *miR-376c* up-regulation needs to be further clarified by adjusting multiple etiological, racial or clinical factors.

It is worth noting that our preliminary assays also show that *miR-376c* is up-regulated in the urine of GC patients. An ROC analysis further specified the level of *miR-376c* in urine as a means of identifying the presence of malignant tissue. Since the sampling of urine is more convenient and non-invasive relative to the sampling of plasma [22, 44], this assessment needs to be further extended in order to assist with the development of a simple clinical diagnosis system that can be used for GC screening.

Human chromosome 14q32 locus has been shown to be amplified in head and neck carcinoma cell lines [45] and in GC, especially in metastatic submucosal-invasive GC [46]. Furthermore, the translocation, loss of heterozygosity, and deletion of chromosome 14q32 has been reported in melanoma [47] and prostate cancer [48] and these changes may underlie the silencing of the *miR-376* cluster. Our findings suggest that 14q32 amplification might be a possible reason for the up-regulation of both *miR-376a* and *miR-376c* in GC since they are up-regulated concordantly. Although *miR-376a* expression is high in tumor tissue, it was not found to be elevated in the patients’ blood. This might be due to barriers either affecting the secretion of *miR-376a* from neoplastic cells to their microenvironment or blocking the release of *miR-376a* from tumors into the circulation; this dichotomy needs to be addressed [49–51]. Alternatively, the relatively high abundance of *miR-376a* in the plasma of control individuals needs to be investigated in more detail.

Apart from the potency of plasma *miR-376c* as a means of distinguishing cancer patients from control individuals, the level of plasma *miR-376c* was also found to be up-regulated in tumors at an early stage of their neoplastic development. This implies that *miR-376c* could be used as a marker for early GC tumorigenesis. Although a previous study was unable to identify an increase in *miR-376c* in the serum of dysplastic gastric lesions [26], there remains a need to further explore the expression status of *miR-376c* during the various stages of gastric carcinogenesis. Our analysis verified that even patients carrying relatively better differentiated tumors also displayed an increased level of *miR-376c* in their plasma. These conflicts with a previous study indicating that poorly differentiated tumors had a higher serum level of *miR-376c* than tumors displaying better differentiation [26].

We have also shown that an induction of *miR-376c* expression increases the proliferation, migration and anchorage-independent growth ability of SCM-1 cells; furthermore, an inhibition of *miR-376c* expression repressed these phenotypes. These findings are important clues supporting the hypothesis that *miR-376c* is oncogenic in gastric carcinogenesis. Our *in silico* analysis using databases substantiated the fact that there is a low abundance of the *ARID4A* transcript in GC tissue and cell lines [32]. The reporter activity showed that *miR-376c* directly bound to the 3’-UTR of *ARID4A* gene and repressed the expression of luciferase gene. Furthermore, our functional assays were able to demonstrate that *ARID4A* has suppressor activity in the SCM-1 cell line. Decreased and increased expression levels of *ARID4A* were noted in GC cells following either *miR-376c* induction or inhibition, respectively. These findings, together with the fact that there is a trend of towards opposite expression levels patterns of *miR-376c* and *ARID4A* in tumor tissue samples, suggest the presence of a *miR-376c*-*ARID4A* regulation axis in GC. The clinical findings also suggest that the down-regulation of *ARID4A* in GC tumors may be modulated by various other factors in addition to *miR-376c*.

The survival rate of the late-stage GC patients is much lower than that of early-stage patients [1, 3]. However, the early diagnosis of GC is somewhat impeded by the lack of markers available for population screening. This study provides clues supporting the idea that plasma *miR-376c* may act as a potential marker for early GC. If urinary *miR-376c* can also be further validated, it could become an invaluable early non-invasive marker for GC. Finally, the fact that *ARID4A* down-regulation is driven by *miR-376c* up-regulation strongly suggests that this novel mechanism contributes to GC pathogenesis.

## Supporting information

S1 Fig Plasma level of *miR-376c* as related to age. Whisker plots. (A) Analysis across control and GC patient subgroups divided by median age. (B) Analysis across GC patients and control subgroups divided by median age. The median ages for control individuals (group 3) and GC patients (group2) are years 70 and 57, respectively. +, mean value. Numbers within parenthesis, numbers of cases. Un-paired *t*-test.
(DOCX)

S2 Fig Analysis of *ARID4A* expression using bioinformatic domains. (A) cBioPortal. *ARID1A* (Upper) and *ARID4A* (Lower) mRNA expression in 478 GC tumor tissues (TCGA, Provisional). (B) EMBL-EBI. *ARID1A* (Upper) and *ARID4A* (Lower) transcripts in 11 GC cell lines detected by RNA-sequencing.
(DOCX)

S3 Fig Original uncropped and unadjusted blots of Fig 5B—α-Tubulin blot. Lt lane, Control treatment; Rt lane, *miR-376c* mimic treatment.
(TIF)

S4 Fig Original uncropped and unadjusted blots of Fig 5B—ARID4A blot. Lt lane, Control treatment; Rt lane, *miR-376c* mimic treatment.
(TIF)

S5 Fig Original uncropped and unadjusted blots of Fig 5B—α-Tubulin blot. Lt lane, Control treatment; Rt lane, *miR-376c* LNA treatment.
(TIF)

S6 Fig Original uncropped and unadjusted blots of Fig 5B–ARID4A blot. Lt lane, Control treatment; Rt lane, *miR-376c* LNA treatment.
(TIF)

S7 Fig Original uncropped and unadjusted blots of Fig 5C–GAPDH blot. Lt lane, siControl treatment; Rt lane, si*ARID4A* treatment.
(TIF)

S8 Fig Original uncropped and unadjusted blots of Fig 5C–ARID4A blot. Lt lane, siControl treatment; Rt lane, si*ARID4A* treatment.
(TIF)

S1 Table *miR-376c* alterations in gastric carcinoma documented in cBioPortal database. (DOCX)

S2 Table The clinical parameters and the qRT-PCR analysis of group 1 samples. (DOCX)

S3 Table The clinical parameters and the qRT-PCR analysis of group 2 samples. (DOCX)

S4 Table The clinical parameters and the qRT-PCR analysis of group 3 samples. (DOCX)

S5 Table The plasma *miR-376c* expression in eight paired pre-surgical and post-surgical samples. (DOCX)

S6 Table The urinary*miR-376c* expression in 11 healthy controls and 20 GC patients. (DOCX)

S7 Table The clinical parameters, *miR-376* and *ARID4A* △△Ct values in 18 pairs of GC and NCM tissues. (DOCX)
