# Supplementary figures and images for: miR-376c promotes carcinogenesis and serves as a plasma marker for gastric carcinoma
Source: PLoS One. 2017 May 9;12(5):e0177346. doi: 10.1371/journal.pone.0177346 (PMC5423644; doi:10.1371/journal.pone.0177346)

**S1 Fig. Plasma level of *miR-376c* as related to age.**

**
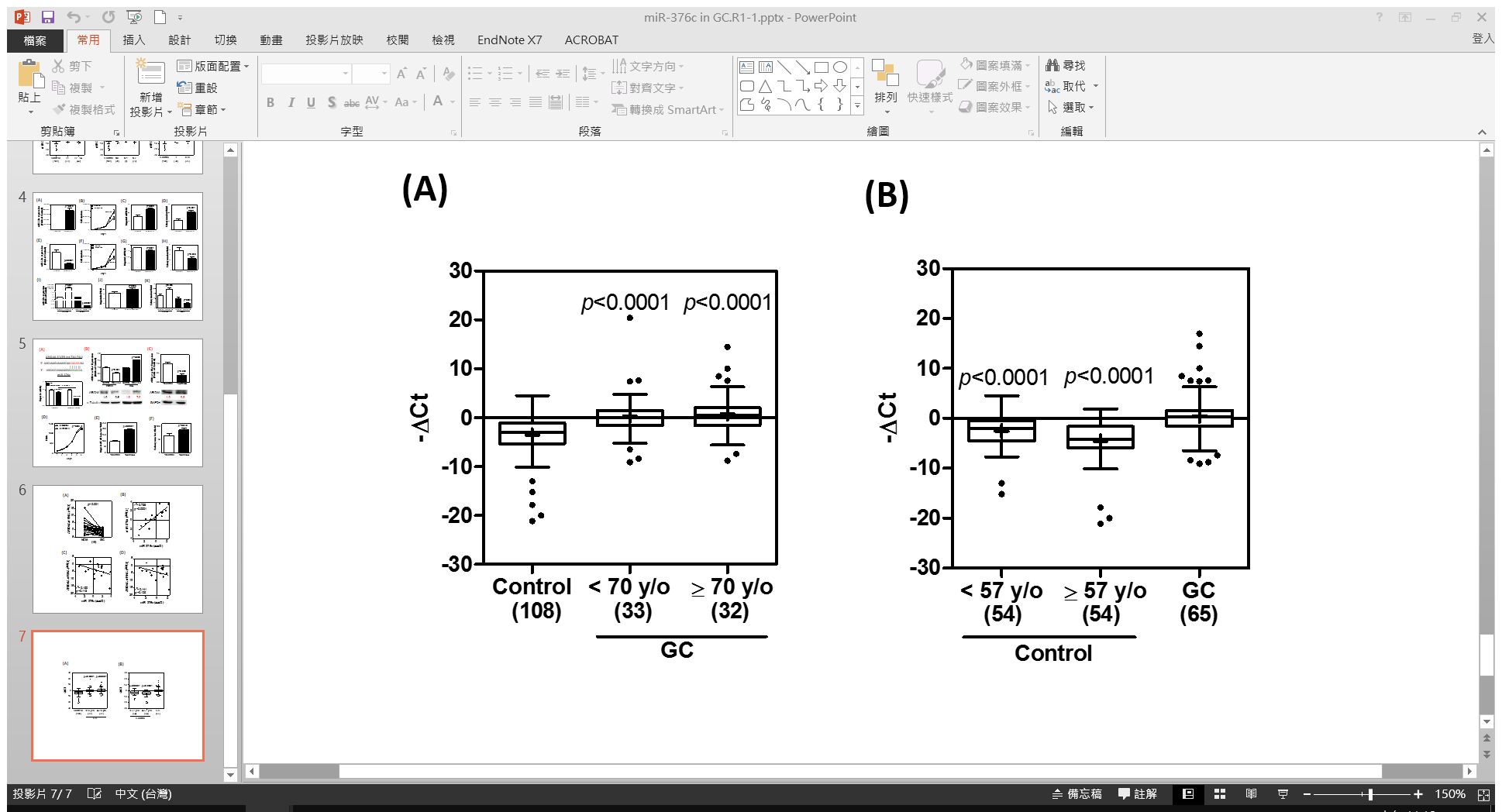
**

Supplement: S1 Fig — Whisker plots. (A) Analysis across control and GC patient subgroups divided by median age. (B) Analysis across GC patients and control subgroups divided by median age. The median ages for control individuals (group 3) and GC patients (group2) are years 70 and 57, respectively. +, mean value. Numbers within parenthesis, numbers of cases. Un-paired t-test. (DOCX) [file pone.0177346.s001.docx]

**S2 Fig. Analysis of *ARID4A* expression using bioinformatic domains.**


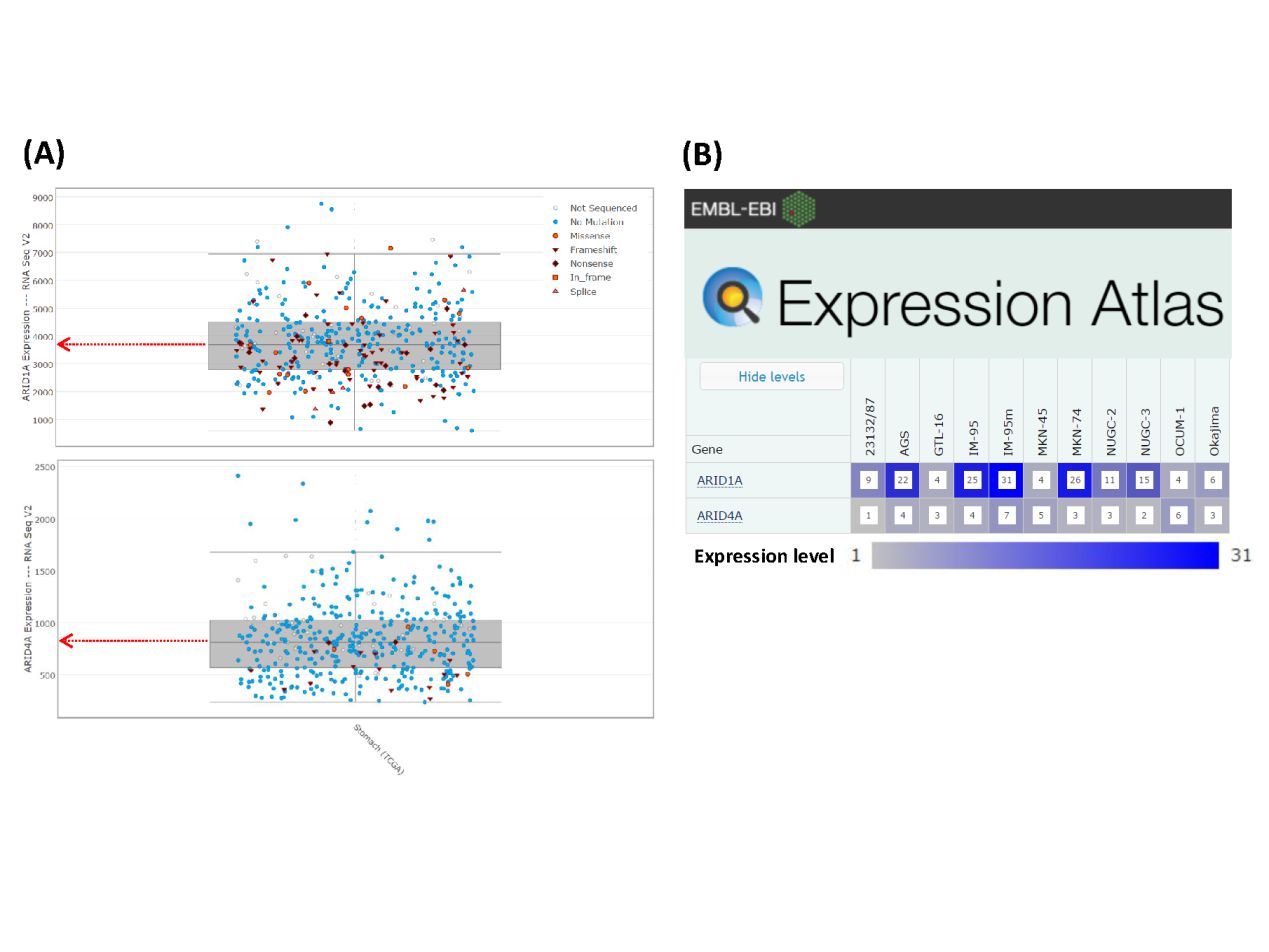

Supplement: S2 Fig — (A) cBioPortal. ARID1A (Upper) and ARID4A (Lower) mRNA expression in 478 GC tumor tissues (TCGA, Provisional). (B) EMBL-EBI. ARID1A (Upper) and ARID4A (Lower) transcripts in 11 GC cell lines detected by RNA-sequencing. (DOCX) [file pone.0177346.s002.docx]

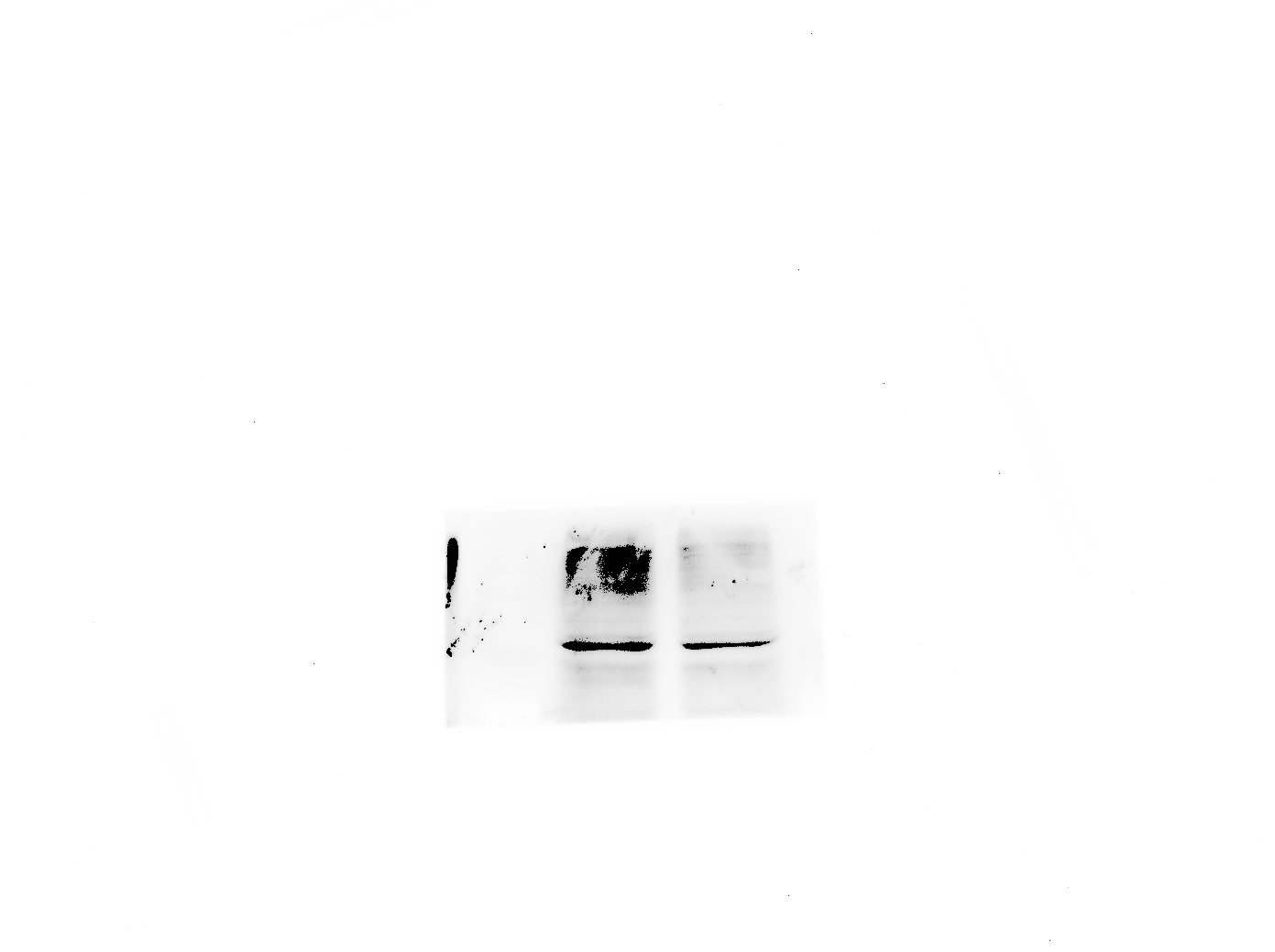

Supplement: S3 Fig — Lt lane, Control treatment; Rt lane, miR-376c mimic treatment. (TIF) [file pone.0177346.s003.tif]

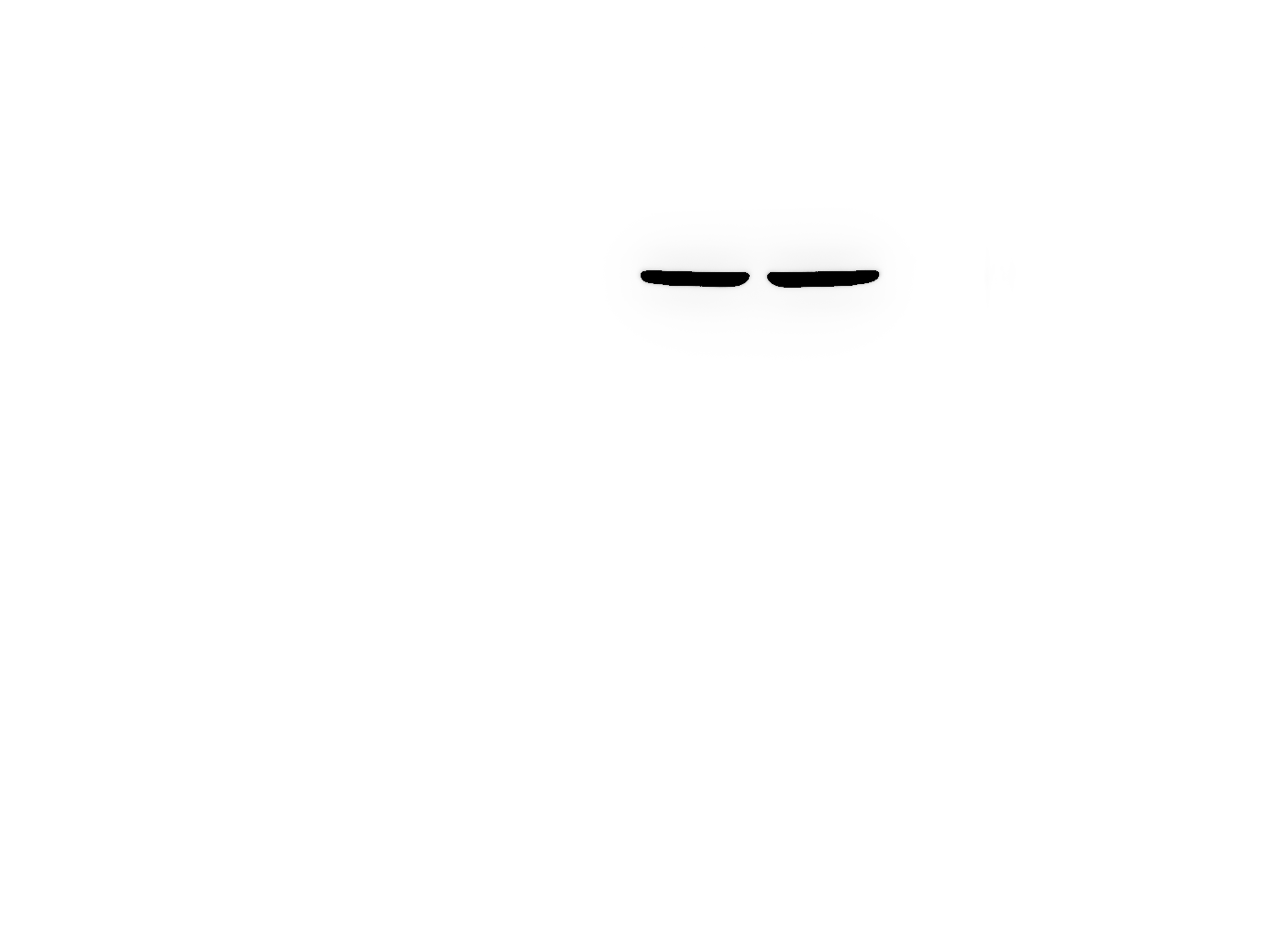

Supplement: S4 Fig — Lt lane, Control treatment; Rt lane, miR-376c mimic treatment. (TIF) [file pone.0177346.s004.tif]

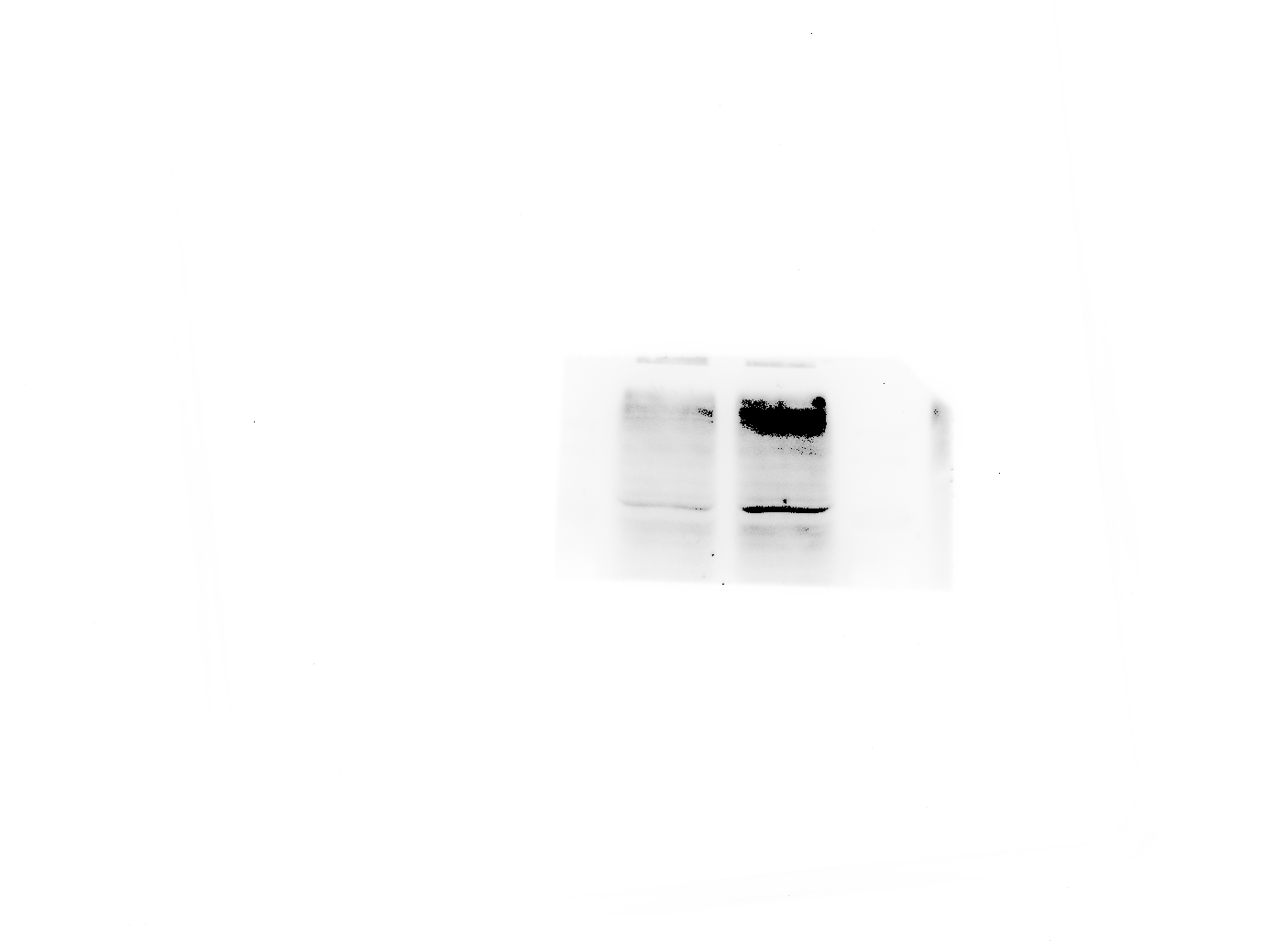

Supplement: S5 Fig — Lt lane, Control treatment; Rt lane, miR-376c LNA treatment. (TIF) [file pone.0177346.s005.tif]

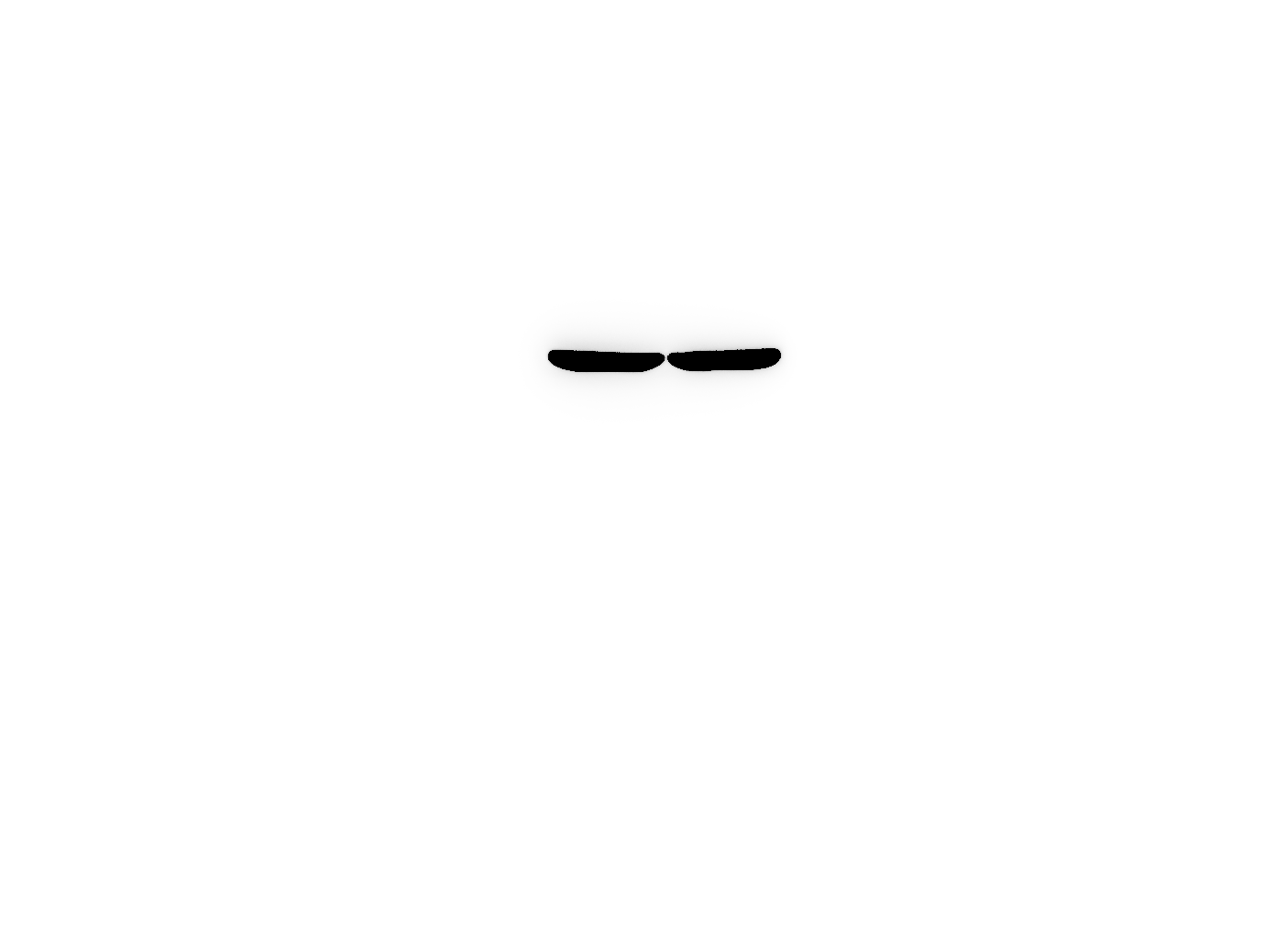

Supplement: S6 Fig — Lt lane, Control treatment; Rt lane, miR-376c LNA treatment. (TIF) [file pone.0177346.s006.tif]

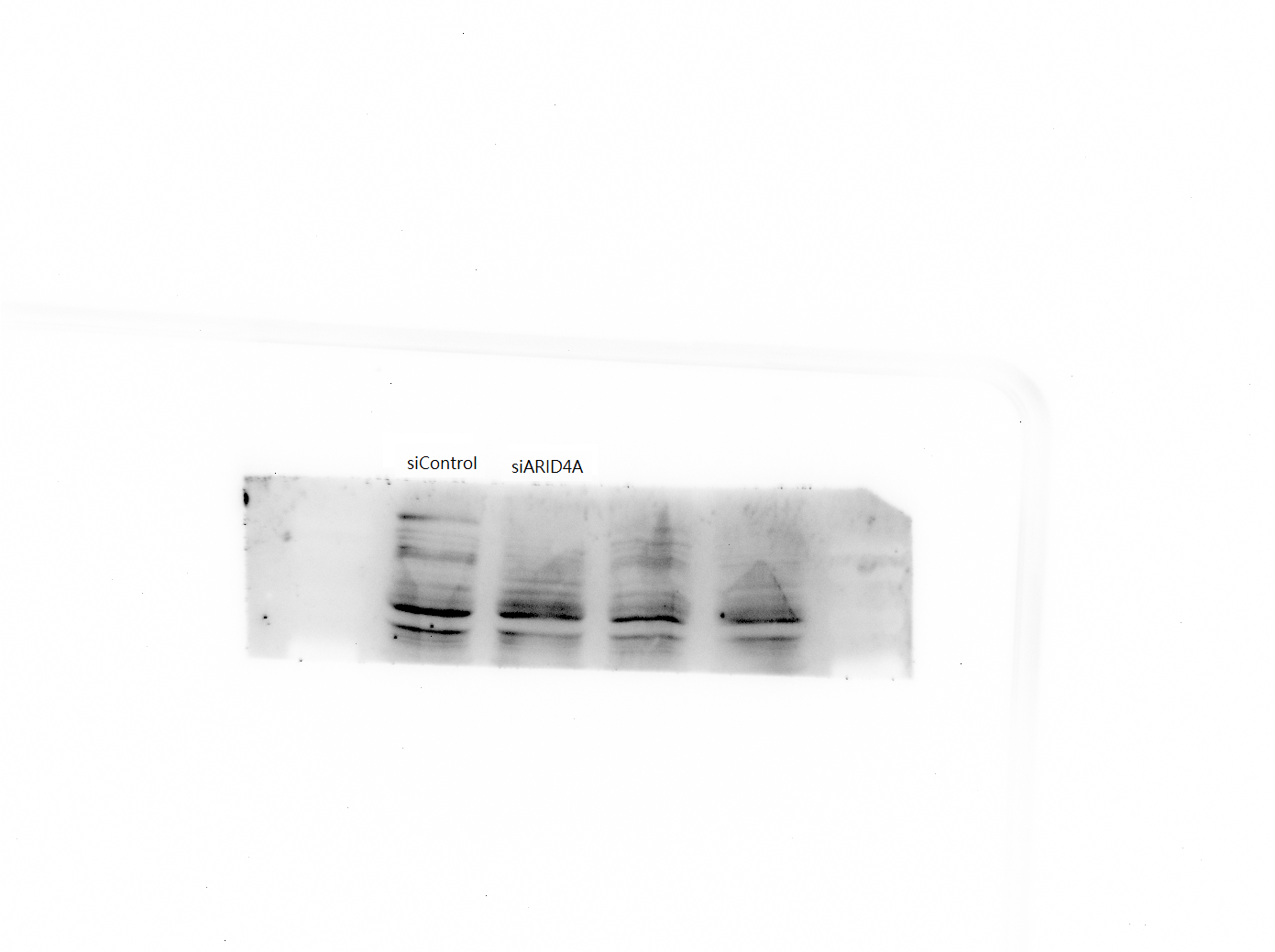

Supplement: S7 Fig — Lt lane, siControl treatment; Rt lane, siARID4A treatment. (TIF) [file pone.0177346.s007.tif]

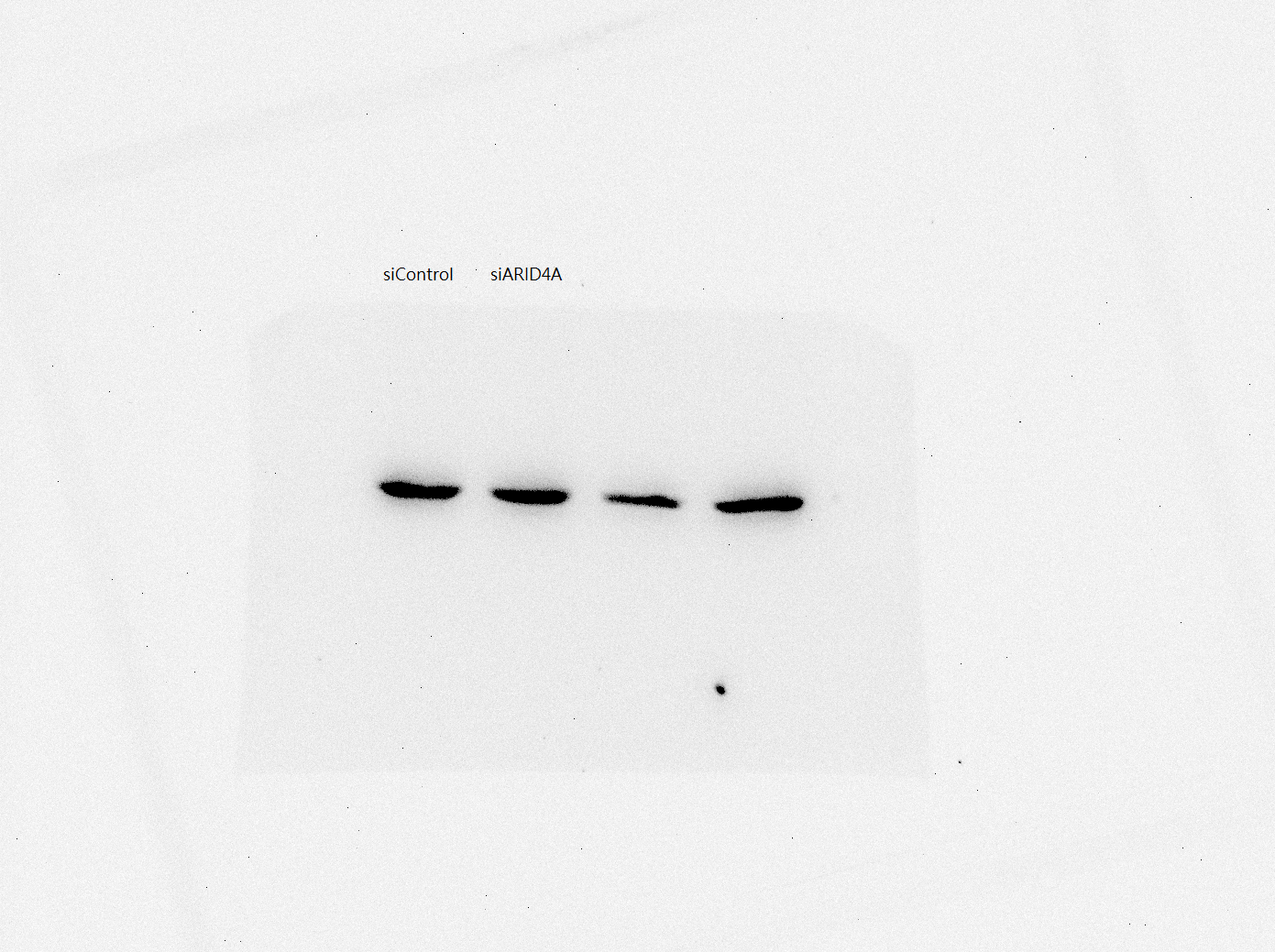

Supplement: S8 Fig — Lt lane, siControl treatment; Rt lane, siARID4A treatment. (TIF) [file pone.0177346.s008.tif]
