# Supplementary material for: miR-376c promotes carcinogenesis and serves as a plasma marker for gastric carcinoma
Source: PLoS One. 2017 May 9;12(5):e0177346. doi: 10.1371/journal.pone.0177346 (PMC5423644; doi:10.1371/journal.pone.0177346)
Supplement: S1 Table — (DOCX) [file pone.0177346.s009.docx]

**S1 Table. *miR-376c* alterations in gastric carcinoma documented in cBioPortal database.**

| **Study name** | **Genetic alteration** | **Summary** |
| --- | --- | --- |
| UHK,  Nat Genet 2011 | Mutation: 0% (0/22) | Exome sequencing of 22 gastric adenocarcinoma samples |
| Pfizer and UHK,  Nat Genet 2014 | Mutation: 0% (0/100) | Whole genome sequencing of 100 tumor-normal pairs from the U. of Hong Kong and Pfizer |
| UTK,  Nat Genet 2014 | Mutation: 0% (0/30) | Whole exome sequencing of 30 diffuse-type gastric adenocarcinoma samples from the U. of Tokyo |
| TCGA, Provisional | 1. Deep deletion: 0.27% (1/369) 2. mRNA up-regulation: 1.08% (4/369) | TCGA Stomach Adenocarcinoma;  raw data at the NCI |
| TGCA,  Nature 2014 | 1. Deep deletion: 0.39% (1/258) 2. mRNA up-regulation: 2.71% (7/258) | TCGA Stomach Adenocarcinoma;  raw data at the NCI |
