# Supplementary material for: miR-376c promotes carcinogenesis and serves as a plasma marker for gastric carcinoma
Source: PLoS One. 2017 May 9;12(5):e0177346. doi: 10.1371/journal.pone.0177346 (PMC5423644; doi:10.1371/journal.pone.0177346)
Supplement: S4 Table — (DOCX) [file pone.0177346.s012.docx]

**S4 Table. The clinical parameters and the qRT-PCR analysis of group 3 samples.**
