# Supplementary material for: miR-376c promotes carcinogenesis and serves as a plasma marker for gastric carcinoma
Source: PLoS One. 2017 May 9;12(5):e0177346. doi: 10.1371/journal.pone.0177346 (PMC5423644; doi:10.1371/journal.pone.0177346)
Supplement: S6 Table — (DOCX) [file pone.0177346.s014.docx]

**S6 Table. The urinary*miR-376c* expression in 11 healthy controls and 20 GC patients.**
