# Supplementary material for: miR-376c promotes carcinogenesis and serves as a plasma marker for gastric carcinoma
Source: PLoS One. 2017 May 9;12(5):e0177346. doi: 10.1371/journal.pone.0177346 (PMC5423644; doi:10.1371/journal.pone.0177346)
Supplement: S7 Table — (DOCX) [file pone.0177346.s015.docx]

**S7 Table. The clinical parameters, *miR-376* and *ARID4A* △△Ct values in 18 pairs of GC and NCM tissues**.
